# Just the facts: What drugs are safe and effective for COVID-19?

**DOI:** 10.1017/cem.2020.403

**Published:** 2020-05-22

**Authors:** Brit Long, Stephen Y. Liang, Hans Rosenberg, Christopher Hicks, Michael Gottlieb

**Affiliations:** * Brooke Army Medical Center, Department of Emergency Medicine, Fort Sam Houston, Texas; † Divisions of Emergency Medicine and Infectious Diseases, Washington University School of Medicine, St. Louis, Missouri; ‡ Department of Emergency Medicine, University of Ottawa, ON; § Division of Emergency Medicine, Department of Medicine, University of Toronto, Toronto, ON; ¶ Department of Emergency Medicine, Rush University Medical Center, Chicago, Illinois

**Keywords:** COVID-19, medications, therapeutics

## Abstract

A 53-year-old male presents with cough, fever, and myalgias for 7 days. Vitals include temperature, 38.0°C; heart rate, 110; blood pressure, 118/70 mm Hg; respiration rate, 28; and oxygen saturation 83% on room air. His only past medical history is hypertension. Your community is in the midst of the coronavirus disease 2019 (COVID-19) pandemic. The patient is hypoxic but responds to oxygen supplementation with nasal cannula and a face mask. His chest x-ray demonstrates multifocal infiltrates. Are there any therapeutic agents currently available for COVID-19?

## CLINICAL SCENARIO

A 53-year-old male presents with cough, fever, and myalgias for 7 days. Vitals include temperature, 38.0°C; heart rate, 110; blood pressure, 118/70 mm Hg; respiration rate, 28; and oxygen saturation 83% on room air. His only past medical history is hypertension. Your community is in the midst of the coronavirus disease 2019 (COVID-19) pandemic. The patient is hypoxic but responds to oxygen supplementation with nasal cannula and a face mask. His chest x-ray demonstrates multifocal infiltrates. Are there any therapeutic agents currently available for COVID-19?

## KEY CLINICAL QUESTIONS

1.: **What is the evidence behind protease inhibitors?**

This class includes lopinavir/ritonavir, which is primarily used for human immunodeficiency virus (HIV) infection. Both medications are protease inhibitors, but ritonavir increases lopinavir concentrations and also prolongs its half-life, serving as a “booster.” Studies have suggested that lopinavir/ritonavir reduces viral load, but there are no definitive data suggesting improved patient outcomes.^1,2^ Lopinavir/ritonavir increases the risk of gastrointestinal adverse effects, can result in QT prolongation, and has a significant number of drug interactions.^2,3^ With no definitive evidence demonstrating improved outcomes in patients with severe infection and adverse events, further data are needed before routine use.^2^

2.: **What is the evidence behind antimalarials?**

This class includes chloroquine and hydroxychloroquine, currently used for autoimmune diseases and malaria treatment and prophylaxis. These medications alter endosomal pH and interfere with viral replication, while chloroquine can also suppress tumor necrosis factor alpha and interleukin-6 (IL-6).^1,2^ These medications may reduce viral load and improve patient outcomes based on one study from China with 100 patients.^S4^ Early studies from France suggested benefit with hydroxychloroquine in patients with severe COVID-19, but the extensive weaknesses in study design, including significant bias, lack of adequate controls, and poor blinding, among many others, severely limit the clinical implications.^2,S5,S6^ A similar subsequent case series found no evidence of antiviral activity or clinical benefit of the combination of hydroxychloroquine and azithromycin for the treatment of hospitalized patients with severe COVID-19^S7^, and two more recent studies of patients with COVID-19 found higher risk of death with use of hydroxychloroquine or chloroquine.^S8, S9^

While hydroxychloroquine is safer than chloroquine, both are toxic in overdose and can cause dysrhythmias and death. While the US Food and Drug Administration (FDA) previously granted authorization to treat COVID-19 with hydroxychloroquine, current guidelines state this medication should only be used in conjunction with a clinical trial.^2,S10^ There are well over 100 trials registered at clinicaltrials.gov currently evaluating these medications. At this time, routine use is not recommended, and the medications may lead to harm.

3.: **When are antibiotics recommended?**

Patients with moderate to severe COVID-19 typically resemble those with sepsis and community-acquired pneumonia. Due to the difficulty in differentiating pneumonia from COVID-19, we recommend use of empiric antibiotics in admitted patients with moderate to severe COVID-19.^S11^ Clinicians should administer agents based on their local antibiogram, but azithromycin combined with an agent that covers *Streptococcus pneumoniae* (e.g., ceftriaxone) is reasonable. Azithromycin may have immunomodulatory and antiviral effects, but it can also result in prolongation of the QT interval.

4.: **What is the evidence behind nucleotide and nucleoside analogs for COVID-19?**

Nucleotide analogs interfere with RNA-dependent RNA polymerases. Remdesivir is an experimental drug that has been studied for use in several viruses.^1,2^ An industry-sponsored case series of 61 patients found clinical improvement in 36 patients, but significant limitations include no control group, unclear patient selection techniques, and no clear primary endpoint.^S12^ A randomized controlled trial (RCT) of admitted patients with COVID-19 (158 received remdesivir and 79 received placebo) found no difference in clinical improvement and no impact on viral load.^S13^ Importantly, this medication should not be given concurrently with other QT prolonging agents, and at the time of writing, further data are needed before routine use.^2,3^

Nucleoside analogs include favipiravir, which has been studied for use in Ebola and influenza.^1,2^ Similar to nucleotide analogs, further data are needed, and approval status with the FDA and Health Canada should be reviewed before use of remdesivir or favipiravir.

5.: **What is the evidence for biologic agents or convalescent plasma?**

Biologic agents include tocilizumab and sarilumab, which are monoclonal antibodies that act against the receptor for IL-6.^2^ These can reduce the inflammatory response by inhibiting the production of acute phase reactants, particularly in the setting of severe COVID-19 infection and cytokine release syndrome (CRS).^2^ Despite the theoretical benefit, there are currently limited data supporting their use. Side effects include elevated transaminases, neutropenia, gastrointestinal perforation, and infusion reactions. Therefore, these monoclonal antibodies should only be considered in patients with CRS.^2^

Convalescent plasma includes passive immunization by administering plasma from patients who have recovered from COVID-19 to those with severe infection.^2^ A recent systematic review of convalescent plasma in the treatment of COVID-19 including 5 studies and 27 patients suggests convalescent plasma could be a safe, effective therapeutic option with a possible mortality benefit. The review could not determine if the higher survival was due to other treatments.^S14^ Several trials are underway to determine optimal dosing and treatment. Convalescent plasma is not recommended for routine use at this time.

6.: **Are medications affecting angiotensin converting enzyme 2 (ACE2) safe in COVID-19?**

SARS-CoV-2 is thought to bind to the ACE2 receptor. Nonsteroidal anti-inflammatory drugs (NSAIDs) and renin-angiotensin-aldosterone system (RAAS) antagonists (e.g., angiotensin-converting enzyme (ACE) inhibitors and angiotensin receptor blockers) may increase ACE2 expression. There are currently no data suggesting patients using these medications are at greater risk of poor outcome with COVID-19. The FDA does not recommend against the use of NSAIDs.^S15^ Regarding RAAS, the American College of Cardiology, American Heart Association, and Heart Failure Society of America state these agents should not be discontinued, and the patient's clinical condition should be considered before modifying a long-term therapeutic regimen.^S16^

## CASE RESOLUTION

**Figure 1.**
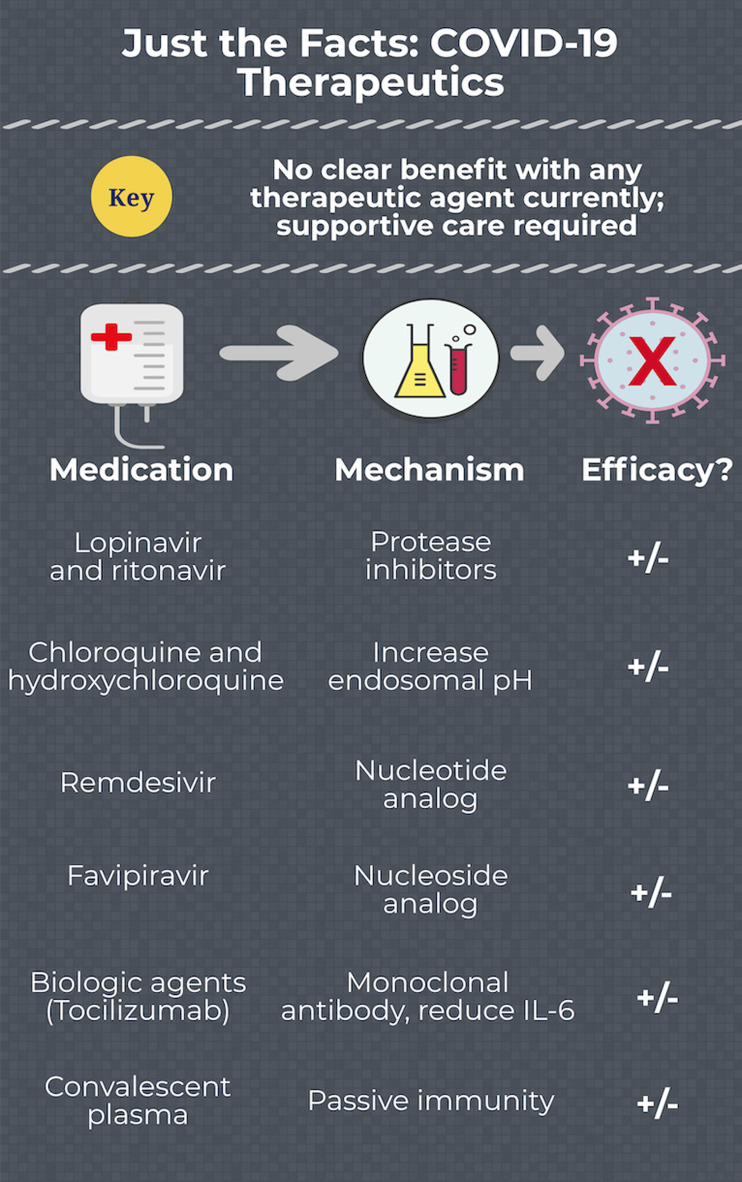
COVID-19 therapeutics.

There are no approved therapeutics for COVID-19 (Figure 1). Many recommendations are extrapolated from severe acute respiratory syndrome coronavirus – 1 (SARS-CoV-1) and Middle East respiratory syndrome coronavirus (MERS-CoV). The literature evaluating therapeutics specifically for COVID-19 suffers from extensive limitations, including the lack of a comparator group, selection bias, industry sponsorship, and very few studies of patient-centered outcomes. Many trials are underway (Table 1), which may assist our management of COVID-19 in the near future.

**Table 1.** Studies currently underway registered at clinicaltrials.gov for therapies reviewed in this article (accessed May 8, 2020)

Therapy	Registered trials
Lopinavir/ritonavir	54
Hydroxychloroquine/chloroquine	265
Remdesivir	21
Favipiravir	14
Convalescent plasma	61
Tocilizumab	41
Sarilumab	13

**KEY POINTS**

1. Apart from supportive care, there are no current effective therapeutics for COVID-19.
2. Most of the studies evaluating therapeutics possess significant limitations.
3. Medications under study include nucleotide and nucleoside analogs, protease inhibitors, antimalarials, convalescent plasma, and biologic agents.
4. There are no data suggesting harm with NSAIDS and RAAS antagonists.

## Supplementary material

For supplementary material accompanying this paper visit http://dx.doi.org/10.1017/cem.2020.403. click here to view supplementary material
