# Supplementary material for: Just the facts: What drugs are safe and effective for COVID-19?
Source: CJEM. 2020 May 22:1–4. doi: 10.1017/cem.2020.403 (PMC7267102; doi:10.1017/cem.2020.403)
Supplement: Supplementary file 1 [file S1481803520004030sup001.docx]

**REFERENCES**

1. Guo YR, Cao QD, Hong ZS, et al. The origin, transmission and clinical therapies on coronavirus disease 2019 (COVID-19) outbreak - an update on the status. Mil Med Res. 2020 Mar 13;7(1):11.

2. Bhimraj A, Morgan RL, Shumaker AH, et al. Infectious Diseases Society of America Guidelines on the Treatment and Management of Patients with COVID-19 Infection. Published April 11, 2020. Available at <https://www.idsociety.org/practice-guideline/covid-19-guideline-treatment-and-management/>

3. Liverpool Drug Interactions Group. Interactions with Experimental COVID-19 Therapies. Available at https://www.covid19-druginteractions.org. 2020 Mar 12.

S4. Gao J, Tian Z, Yang X. Breakthrough: Chloroquine phosphate has shown apparent efficacy in treatment of COVID-19 associated pneumonia in clinical studies. Biosci Trends. 2020 Mar 16;14(1):72-73.

S5. Gautret P, Lagier JC, Parola P, et al. Hydroxychloroquine and azithromycin as a treatment of COVID-19: results of an open-label non-randomized clinical trial. Int J Antimicrob Agents. 2020 Mar 20.

S6. Gautret P, Lagier J, Parola P, et al. Clinical and microbiological effect of a combination of hydroxychloroquine and azithromycin in 80 COVID-19 patients with at least a six-day follow up: an observational study. Available at <https://www.mediterranee-infection.com/wp-content/uploads/2020/03/COVID-IHU-2-1.pdf>.

S7. Molina JM, Delaugerre C, Goff JL, et al. No Evidence of Rapid Antiviral Clearance or Clinical Benefit with the Combination of Hydroxychloroquine and Azithromycin in Patients with Severe COVID-19 Infection [published online ahead of print, 2020 Mar 30]. Med Mal Infect. 2020;S0399-077X(20)30085-8. doi:10.1016/j.medmal.2020.03.006

S8. Magagnoli J, Narendran S, Pereira F, et al. Outcomes of hydroxychloroquine usage in United States veterans hospitalized with Covid-19. 2020. doi: https://doi.org/10.1101/2020.04.16.20065920

S9. Borba MGS, Val FFA, Sampaio VS, et al. Chloroquine diphosphate in two different dosages as adjunctive therapy of hospitalized patients with severe respiratory syndrome in the context of coronavirus (SARS-CoV-2) infection: Preliminary safety results of a randomized, double-blinded, phase IIb clinical trial (CloroCovid-19 Study). 2020. doi: https://doi.org/10.1101/2020.04.07.20056424

S10. U.S. Food and Drug Administration. Request for Emergency Use Authorization For Use of Chloroquine Phosphate or Hydroxychloroquine Sulfate Supplied From the Strategic National Stockpile for Treatment of 2019 Coronavirus Disease. Available at <https://www.fda.gov/media/136534/download>. Accessed April 10, 2020.

S11. Alhazzani W, Møller, MH, Arabi YM, et al. Surviving Sepsis Campaign: Guidelines on the Management of Critically Ill Adults with Coronavirus Disease 2019 (COVID-19).

S12. Grein J, Ohmagari N, Shin D, Diaz G. Compassionate Use of Remdesivir for Patients with Severe Covid-19. N Engl Journ Med. Published April 10, 2020. DOI: 10.1056/NEJMoa2007016

S13. Wang Y, Zhang D, Du G, et al. Remdesivir in adults with severe COVID-19: a randomised, double-blind, placebo-controlled, multicentre trial. The Lancet. April 2020. doi:[10.1016/s0140-6736(20)31022-9](https://doi.org/10.1016/s0140-6736(20)31022-9)

S14. Rajendran K, Narayanasamy K, Rangarajan J, Rathinam J, Natarajan M, Ramachandran A. Convalescent plasma transfusion for the treatment of COVID-19: Systematic review [published online ahead of print, 2020 May 1]. J Med Virol. 2020;10.1002/jmv.25961. doi:10.1002/jmv.25961

S15. U.S. Food and Drug Administration. FDA advises patients on use of non-steroidal anti-inflammatory drugs (NSAIDs) for COVID-19. Published March 19, 2020. Available at <https://www.fda.gov/drugs/drug-safety-and-availability/fda-advises-patients-use-non-steroidal-anti-inflammatory-drugs-nsaids-covid-19>. Accessed April 8, 2020.

S16. American College of Cardiology. HFSA/ACC/AHA Statement Addresses Concerns Re: Using RAAS Antagonists in COVID-19-ACC. Published March 17, 2020. Available at https://www.acc.org/latest-in-cardiology/articles/2020/03/17/08/59/hfsa-acc-aha-statement-addressesconcerns-re-using-raas-antagonists-in-covid-19. Accessed March 29, 2020.
